# Junctioning longitudinally adjacent PTVs with Helical TomoTherapy

**DOI:** 10.1120/jacmp.v11i2.3047

**Published:** 2010-04-16

**Authors:** Lourdes M. Garcia, Lee H. Gerig, Peter Raaphorst, David Wilkins

**Affiliations:** ^1^ Department of Physics Carleton University Ottawa Canada K1S5B6; ^2^ Department of Medical Physics The Ottawa Hospital Cancer Centre Ottawa Canada K1H8L6

**Keywords:** PTV junctioning, helical tomotherapy

## Abstract

Irradiation of longitudinally adjacent PTVs with Helical TomoTherapy (HT) may be clinically necessary, for example in treating a recurrent PTV adjacent to a previously‐treated volume. In this work, the parameters which influence the cumulative dose distribution resulting from treating longitudinally adjacent PTVs are examined, including field width, pitch, and PTV location. In‐phantom dose distributions were calculated for various on‐ and off‐axis cylindrical PTVs and were verified by ion chamber and film measurement. Dose distributions were calculated to cover 95% of the PTV by the prescribed dose $\left(D_{P}\right)$ using 25 and 50 mm long HT fields with pitches of either 0.3 or 0.45. These dose distributions where then used to calculate the 3D dose distribution in the junction region between two PTVs. The best junction uniformity was obtained for fields of equal width, with larger fields providing better intra‐PTV dose homogeneity than smaller fields. Junctioning fields of different widths resulted in a much larger dose inhomogeneity, but this could be improved significantly by dividing the junction end of the PTV treated with the smaller field into multiple (up to 4) sub‐PTVs, with the prescribed dose in each sub‐PTV decreasing with proximity to the junction region. This provided a PTV matching with dose homogeneity similar to that achieved when junctioning two PTVs, both irradiated by the 50 mm field, and provided a distribution where 95% of the PTV received at least the prescribed dose, with maximum excursions from prescribed dose varying from −19% to +13%. We conclude that junctioning adjacent PTVs is possible. Treating longitudinally adjacent PTVs with different widths is a challenge, but dose uniformity is improved by breaking PTVs into multiple contiguous sub‐PTVs modified to feather (broaden) the effective junctioning region.

PACS number: 87.55.D

## I. INTRODUCTION

Helical TomoTherapy (HT) is a relatively new radiation therapy technology that can deliver highly conformal dose distributions to complex target shapes while reducing the dose to critical normal tissues. It is an accepted treatment modality for many cancers such as head and neck,^(^ ^1^ ^–^ ^9^ ^)^ prostate,^(^ ^10^ ^–^ ^12^ ^)^ spinal metastasis,^(13)^ and CNS,^(4)^ where the ability to plan and deliver highly conformal complex dose distributions under image guidance is thought to hold benefit.

There arise clinical situations where it is necessary or of benefit to junction two different longitudinally adjacent treatment volumes to form a contiguous treated volume (PTV). A common clinical example is the need to treat a recurrence (or new lesion) PTV that is just superior or inferior to a previously treated PTV. Another example, as important but less obvious, is where significant clinical gain can be achieved by treating different longitudinally adjacent volumes with different field widths. Such a case could arise where the transverse cross section in one portion of the PTV varies slowly in the SUP‐INF direction but, in another region, the transverse cross section varies rapidly with longitudinal position. The former would lend itself to treatment by a large field, while the later would receive a more conformal distribution (reduced NTCP) if treated by a smaller HT field width. With the existing TomoTherapy unit, this requires planning two separate PTVs, each with a different beam, to form one contiguous dose volume. TomoTherapy also limits the bed travel to 160 cm; so for treatments such as total marrow irradiation^(^ ^14^ ^–^ ^17^ ^)^ of tall patients, two separate adjacent PTVs must be planned and treated to form a contiguous volume.

In this work, we examine the dosimetric issues of field junctioning with TomoTherapy, and the influence that parameters such as field width, pitch, and PTV location have on the integral dose distribution.

## II. MATERIALS AND METHODS

HT has a design similar to a modern CT scanner, in which a 6 MV linac, mounted on a ring gantry, continuously rotates with the central axis of the MV beam intersecting the axis of rotation of the gantry, while the patient treatment couch moves with a constant velocity through the gantry bore and parallel to gantry axis of rotation, providing a helical radiation delivery pattern.^(18)^ ^,^ ^(19)^

The unfiltered 6 MV radiation beam is collimated to produce a rotating fan beam, with the long axis perpendicular to the axis of rotation. The fan‐beam width at isocentre (85 cm) has three preset values of 10, 25 and 50 mm (field widths). The beam is further collimated by a multileaf collimator of 64 leaves allowing a maximum field size of 40 cm in the transverse direction. Pitch, another delivery parameter studied in this work, is defined as the ratio of the distance that the couch moves per gantry rotation divided by the field width.^(17)^

The results presented in this work are based on planning studies, but the predicted dose distributions from each individual field were verified by ion chamber and film dosimetry.

Integral dose distributions resulting from the junctioning of identical and different fields are calculated by summing individual dose distributions separated by different spacing. The study considers adjacent fields of different pitches and field widths, and also examines cases where the volumes of interest are at different distances from the gantry axis. Cumulative and differential dose volume histograms were calculated and analyzed.

### A. Geometry and setup

**Figure 1:**
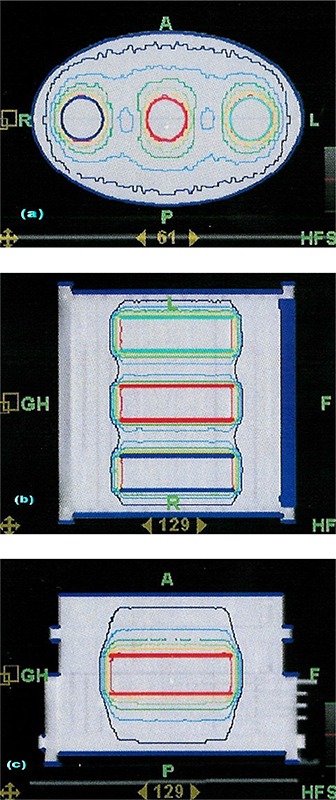
Axial (a), coronal (b) and sagittal (c) views of the phantom, PTVs, and isodose distribution calculated by the TPS for 25 mm field and pitch of 0.3.

Planning and measurements were performed on a pseudo‐anthropomorphic homogeneous phantom, consisting of an acrylic (PMMA) elliptical (major axis 30 cm, minor axis 20 cm) cylinder (27 cm long). Within the phantom, three parallel cylindrical 14.4 cm long PTVs are defined. The axis of each PTV is parallel to the y‐axis (IEC 1217^(20)^) of the HT machine with one being on the y‐axis (on‐axis, X = 0), and the other two being off‐axis at X equal +9.4(left) and −9.4cm(right). There was a 6 cm margin between the caudal and cranial edges of the phantom and INF and SUP borders of the PTVs respectively. The axial, sagittal and coronal views of the phantom and the PTVs are shown in Fig. 1. A TomoTherapy Hi·Art System (TomoTherapy Inc., Madison, WI, USA) was used for the planning, optimization and dose delivery.

The first experimental step in this work was to dosimetrically verify the dose distributions predicted by planning system. Experiments consisted of creating plans to deliver 4 Gy per fraction to each PTV for each of the three available field widths. The treatment plans were then delivered to the phantom and the resulting dose distributions for single PTV plans were measured with Kodak EDR2 film. A single point in each PTV was measured with an ADL calibrated Standard Imaging A1SL ion chamber and Fluke electrometer (model: KEITHLEY 35040 Therapy Dosimeter).

**Figure 2:**
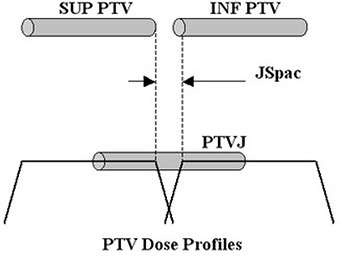
Scheme of PTVJ definition within the junction region.

The calculated 3D dose distributions for each of the different field widths, pitches and off‐axis distances and PTVs were then exported as DICOM images for offline processing in MATLAB R2007a. In this environment and with in‐house software, the 3D junctioned dose distributions were calculated as the sum of two dose distributions and then analyzed. This was done by summing two different distributions spatially offset from each other to correspond to the cranial‐caudal (CC) distance between two adjacent PTVs used to form a contiguous volume. This offset distance will be referred to as junction spacing (JSpac). New cylindrical PTVs, referred to as PTVJs, were then defined to contain the volumes of the junctions between the initial PTVs. In order to assess the dose homogeneity and compare to a reference dose distribution, the PTVJs were selected such that the junction region was included (in the middle) and had the same cross‐sectional area and length as the original SUP and INF PTVs shown in Fig. 2. Having all the same volume (PTVs and PTVJs), the DVHs and dose profiles in these PTVJs are compared to reference dose distributions for a single PTV planned and treated with a continuous field.

### B. Treatment planning procedure

The phantom was imaged on a Philips Brilliance CT‐Simulator at 120 KV. The Hounsfield number to physical density (used in HT planning) included a value for PMMA $\left(1.18\;g/{\text{cm}}^{3}\right)$.

A total of 120 slices, 3 mm thick, were acquired. The PTVs were outlined on the Pinnacle^3^ version 7.6c planning station and exported to TomoTherapy Planning Station (TPS). The prescription was 20 Gy in 5 fractions (4Gy/fx) with 95% of the PTVs to be covered by the prescribed dose $\left(D_{P}\right)$. Treatments were designed to deliver a uniform dose to one or multiple PTVs. Field widths of 25 and 50 mm at pitches of 0.3 and 0.45 were examined. An initial modulation factor of 2 was used. The other parameters of the planning process including importance, maximum and minimum doses, DVH dose and penalties were optimized at the beginning and kept constant for all subsequent experiments. Figure 1 shows the axial, coronal and sagittal phantom images; the PTVs and the planned isodose distribution for a 25 mm field and a pitch of 0.3. The 3D dose matrixes calculated by the TPS and the vectors defining the PTV structures were then exported for offline processing in MATLAB.

In order to verify the agreement between the planned and delivered dose, the CC penumbral slopes were confirmed with film dosimetry with films placed in the central coronal plane of the phantom. The slopes were calculated for field widths of 25 and 50 mm, and pitch of 0.3. CC penumbral slopes were determined from central longitudinal dose profiles for on‐/off‐axis PTVs from both measured and planned dose distributions.

## III. RESULTS

### A. Penumbral slope

**Figure 3:**
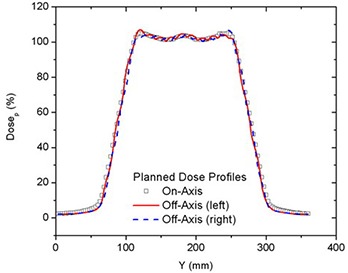
Comparison of on‐ and off‐axis PTVs dose profiles for 50 mm field width for deliveries with a pitch of 0.3.

**Figure 4:**
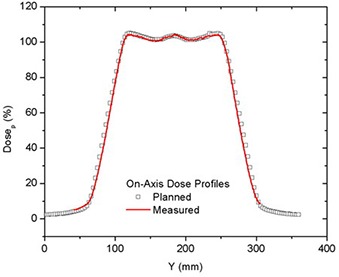
Comparison of on‐axis planned and measured dose profiles for 50 mm field width for deliveries with a pitch of 0.3.

On‐/off‐axis planned dose profiles along the central axis of each PTV are shown in Fig. 3 for a 50 mm field delivered at a pitch of 0.3. A larger thread effect was seen off‐axis than on‐axis.^(21)^ To confirm the TPS dose distributions, the axial profiles from planning were compared to those extracted from coronal film measurements made in the phantom for the same treatment. Typical axial planned and measured dose profiles are shown in Fig. 4 for 50 mm field width and pitch of 0.3. The profiles were normalized to $D_{P}$.

In addition to beam profiles, CC penumbral slopes are also computed and compared. These results are shown in Table 1 for the cases described above. The reported uncertainties are the parametric error from the fit.

**Table 1:** Planned and measured CC penumbral slopes, on‐ and off‐axis, for 50 mm field width and pitch of 0.3.

			*PTV's Penumbral Slope (Gy/mm)*			
*Profiles*	*On‐Axis (Central)*		*Off‐Axis (Right)*		*Off‐Axis (Left)*	
	*Cranial*	*Caudal*	*Cranial*	*Caudal*	*Cranial*	*Caudal*
Planning	0.0798	0.0794	0.083	0.084	0.085	0.083
	±0.0004	±0.0004	±0.002	±0.002	±0.002	±0.002
Measurement	0.0795	0.0793	0.084	0.0816	0.0811	0.083
	±0.0006	±0.0006	±0.001	±0.0008	±0.0008	±0.001
% Diff	0.4	0.1	1.2	2.9	4.8	0.0

The last row on Table 1 gives the percent difference between measured and planned slopes. The agreement between CC penumbral slopes from measurements and planning was better than 5% for all the cases included in this study, and the distance to dose agreement was always less than 3 mm. No statistically significant difference between the cranial and caudal gradients was seen.

### B. Field width, pitch and off‐axis distance effects on dose gradients

CC penumbral dose gradients (%/mm) at 50% of $D_{P}$ were found to be inversely proportional to the field width $\left(\approx D^{P}\times 100\%/\text{field}\;\text{width}\right)$. The dose gradients are shown in Table 2. Slightly larger CC gradients were seen off‐axis than on‐axis. The dose gradients were found to be independent of pitch except as influenced by the thread effect which increases with off‐axis distance, as seen in Fig. 3.^(21)^ Gradients were not affected by simultaneously treating one, two or all three PTVs.

**Table 2:** On‐ and off‐axis dose gradients at 50% of $D_{P}$ for 25 mm and 50 mm fields and pitches of 0.3 and 0.45.

	*Dose Gradients ‐ at 50% of DP* (<±0.1%/mm)			
*Pitch*	*0.3*		*0.45*	
*Field width (mm)*	*50*	*25*	*50*	*25*
On‐axis	2.0	3.8	2.0	3.8
Off‐axis				
Right	2.1	4.1	2.1	4.1
Left	2.1	4.1	2.1	4.1

### C. Matching fields of equal width

**Figure 5:**
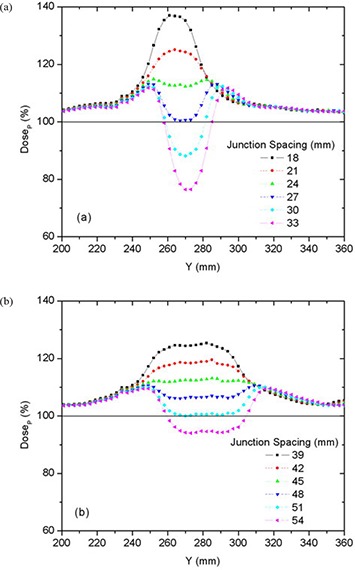
Comparison of on‐axis dose profiles depending on junction spacing resulting from matching fields of equal width of 25 mm (a) or 50 mm (b) for deliveries with a pitch of 0.3.

**Figure 6:**
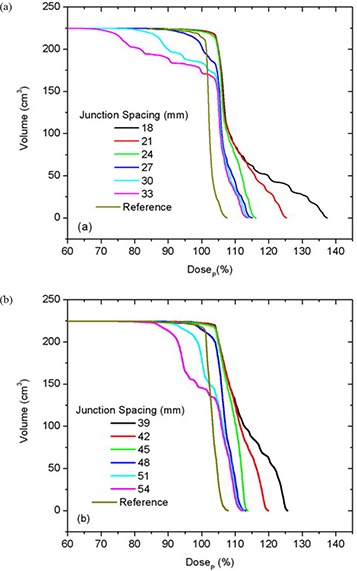
DVHs for on‐axis PTVJ arising from the matching of equal fields of 25 mm (a) and 50 mm (b) for deliveries with a pitch of 0.3. The references represent the DVH for continuous field (no junction).

The best junction uniformity was obtained for fields of equal width. Junctioning of adjacent PTVs treated with a larger field width (e.g. 50 mm) produced more homogeneous junctions than junctions of PTVs treated with smaller field widths (25 mm), as shown in Fig. 5. This figure shows the central axis dose profiles across the junction between adjoining PTVs for different JSpac from matching 25 mm fields (a) and 50 mm fields (b). Only the section of the profiles within in the junction region can be seen in the profiles plots (to better illustrate the dose behavior). The mean dose, standard deviation (SD), second moment about $D_{P}$ (SM), minimum (Min) and maximum (Max) doses to the on‐axis PTVJ as a function of JSpac are shown in Table 3 from matching equal fields of 25 mm or 50 mm. The corresponding values obtained for continuous fields (no junctioning, on‐axis PTV) for 25 mm and 50 mm fields are also shown, for reference (last row of Table 3). Figure 6 shows the corresponding DVHs for on‐axis PTVJ and PTV (reference). Pitch in all examples shown was 0.3.

**Table 3:** On‐axis PTVJ mean, SD, Min and Max doses, and SM depending on junction spacing from matching equal fields of 25 mm or 50 mm, and pitch of 0.3.

*On‐axis PTVJ*							
*Fields of 25 mm*				*Fields of 50 mm*
*JSpac (mm)*	$\text{Mean}/\;\text{SD}\;(\%D_{P})$	$\text{Min‐Max}\;(\%D_{P})$	$\text{SM}\;({\text{Gy}}^{2})$	*JSpac (mm)*	$\text{Mean}/\;\text{SD}\;(\%D_{P})$	$\text{Min‐Max}(\%D_{P})$	$\text{SM}\;({\text{Gy}}^{2})$
18	113/10	88–138	.42	39	113/7	87–126	.37
21	110/7	88–125	.24	42	111/5	87–120	.24
24	108/4	87–116	.13	45	109/3	87–114	.15
27	106/5	77–115	.09	48	107/3	83–113	.09
30	104/8	68–114	.11	51	105/5	78–113	.07
33	102/11	59–114	.20	54	102/7	73–112	.10
Ref	102/2	84–107	.02	Ref	103/2	85–108	.02

Doses in excess of $D_{P}$ were seen in both PTVJs and PTVs. PTVJ average doses, similar to the ones achieved by continuous fields, can be obtained. But this is done at the expense of dose homogeneity within the junction (as determined by an increased of the SD and SM) with minimum dose reaching as low as 59% and 73% of $D_{P}$ for 25 mm and 50 mm fields, respectively (second last column of Table 3).

The optimal JSpac between adjoining PTVs was determined by finding simultaneously the smallest possible SD, which ensures dose uniformity, and small SM to guarantee doses closer to the prescription. SD is frequently used as a measure of the spread and has been reported as an effective homogeneity index of intensity‐modulated radiotherapy plans.^(22)^ However, SD would be the best estimator if the underlying distribution were normal. Normality is not expected for the dose distribution within the junction in the presence of cold (large JSpac) or hot (small JSpac) spots (see Fig. 5), but near the optimal junctioning, SD will be a better estimator. Irrespective of this, the SD can still be used on a relative basis. SM was also calculated to assess the dose variability about $D_{P}$.

For fields of equal width, the optimal JSpac between adjoining PTVs, defined as described above, was 27 mm and 48 mm for fields of 25 mm and 50 mm, respectively. These junction distances ensured that 95% of the on‐axis PTVJ received at least 98% of the $D_{P}$ for 25 mm fields and at least 100% of the $D_{P}$ for 50 mm fields (optimal junction), for a planned objective of 95% of the PTVs and PTVJs receiving 100% of the $D_{P}$.

**Figure 7:**
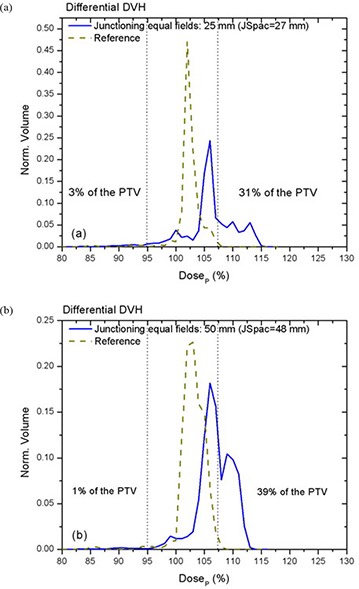
Normalized differential DVHs for on‐axis PTVJ arising from the matching of equal fields (optimal JSpac) are represented by solid lines for fields of 25 mm (a) and 50 mm (b) for deliveries with a pitch of 0.3. Corresponding references for on‐axis PTV are shown in dashed lines.

Figure 7 shows the differential DVH for on‐axis PTVJs from matching equal fields of 25 mm (a) and 50 mm (b) for the optimal spacing. The narrowest differential DVH was achieved using equal fields of 50 mm. Here the dose range extended from −17% to +13% of the prescription, although only 1% of the on‐axis target volume (on‐axis PTVJ) received doses lower than 95% of the $D_{P}$.

### D. Matching fields of different widths

**Figure 8:**
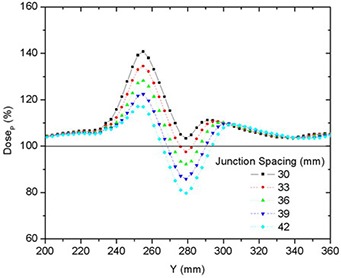
Comparison of on‐axis dose profiles depending on junction spacing resulting from matching fields of different widths of 25 mm and 50 mm for deliveries with a pitch of 0.3.

**Figure 9:**
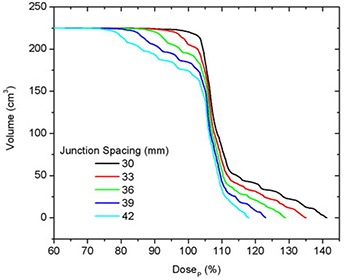
DVHs for on‐axis PTVJ arising from the matching of two different fields of 25 mm and 50 mm for deliveries with a pitch of 0.3.

**Figure 10:**
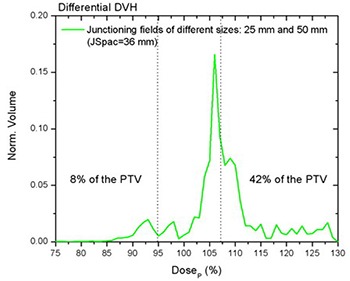
Normalized differential DVH for on‐axis PTVJ arising from the matching of different fields of 25 mm and 50 mm (optimal JSpac) for deliveries with a pitch of 0.3.

Junctioning fields of different widths resulted in a more complex dose distribution than matching fields of equal width. Table 4 shows the on‐axis PTVJ average doses, SD, SM Min and Max doses for different JSpac from matching different fields of 25 mm and 50 mm. Figures 8 and 9 show the dose profiles along the central axis of the on‐axis PTVJ and the corresponding DVH as a function of the JSpac. As can be seen, homogeneous field matching could not be achieved. The minimum volume dose excursion from that prescribed was achieved with a JSpac of about 36 mm, at a cost of underdosing by a maximum of 26% and overdosing by 29%. In this case, 95% of the PTVJ received at least 92% of the $D_{P}$, lower than that obtained for junctions with equal field widths. The normalized differential DVH are shown in Fig. 10. In this case, 8% of the PTVJ received less than 95% of the prescription.

**Table 4:** On‐axis PTVJ mean, SD, Min and Max doses, and SM depending on junction spacing from matching different fields of 25 mm and 50 mm, and pitch of 0.3.

*On‐axis PTVJ Fields of 25 and 50 mm*			
*JSpac (mm)*	$\text{Mean}/\text{SD}(\%D_{P})$	$\text{Min‐Max}(\%D_{P})$	$\text{SM}({\text{Gy}}_{2})$
30	112/10	84–141	.39
33	110/9	80–135	.27
36	107/8	74–129	.19
39	105/9	68–123	.16
42	103/10	63–118	.17
45	101/12	59–115	.22

**Figure 11:**
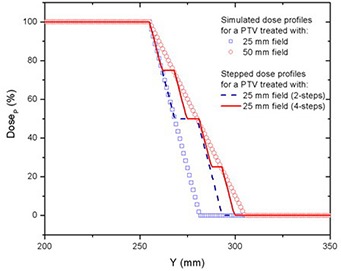
INF end of the PTV simulated profiles for field widths of 25 mm and 50 mm, and the simulated stepped dose profiles from breaking the PTVs into smaller sub‐PTVs for deliveries with 25 mm field width.

In order to reduce the dose heterogeneity, the PTVs were divided into two or four smaller sub‐PTVs near the junction (only for deliveries with a 25 mm field), with the sub‐PTV prescription dose decreasing toward the junction so that the adjacent PTVs had similar CC dose profiles. Simulated profiles based on CC penumbral slopes were first generated to assess the field matching depending on the sub‐PTV dimensions. Figure 11 shows examples of the INF end of the PTV simulated profiles for both field widths (25 mm and 50 mm). The stepped dose profiles from breaking the PTVs into smaller sub‐PTVs are also shown.

**Figure 12:**
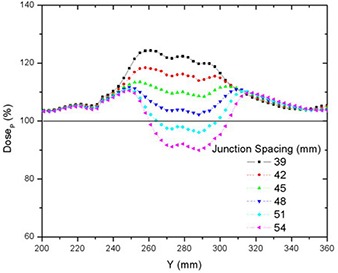
Comparison of on‐axis dose profiles depending on junction spacing resulting from matching fields of different widths of 25 mm (4‐steps) and 50 mm for deliveries with a pitch of 0.3.

**Figure 13:**
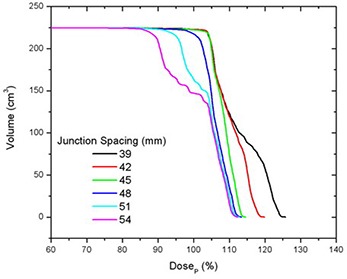
DVHs for on‐axis PTVJ arising from the matching of two different fields of 25 mm (4‐steps) and 50 mm for deliveries with a pitch of 0.3.

**Figure 14:**
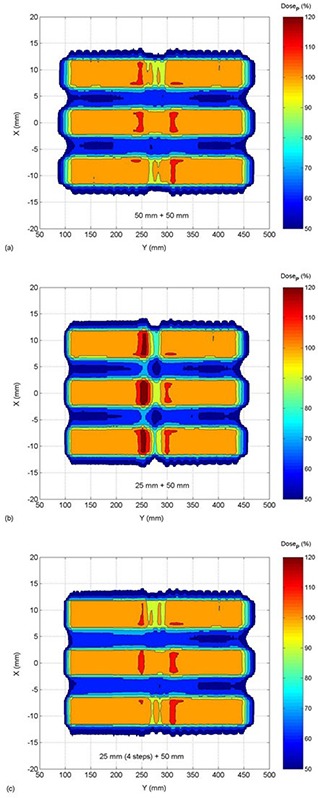
Isodose maps from the matching of equal fields of 50 mm width (a), different fields of 25 mm and 50 mm (b), and different fields of 25 mm (4‐steps) and 50 mm (c). The the dose distribution for the optimal JSpac corresponding to 48 mm (a), 36 mm (b) and 48 mm (c), respectively.

Dividing each PTV into four multiple contiguous sub‐PTVs, with constantly decreasing prescribed dose $\left(D_{P},\;3/4D_{P},\;1/2D_{P},\;1/4D_{P}\right)$ allowed PTV matching with dose homogeneity similar to junctioning PTVs of equal CC slope. Ninety‐five percent of the PTV received at least 101% of the $D_{P}$, with dose excursions of −19% to +13% from prescription (1% of the PTV received less than 95% of $D_{P}$). Summed dose profiles from matching PTVs with fields of different widths of 25 mm (4‐steps) and 50 mm depending on junction spacing are shown in Fig. 12. The corresponding DVHs are seen in Fig. 13. For comparison, the isodose curves for three different cases are shown in Fig. 14: matching of equal fields of 50 mm width (a), different fields of 25 mm and 50 mm (b), and different fields of 25 mm (4‐steps) and 50 mm (c). In each case, the figures represent the dose distribution arising from optimal inter‐PTV spacing (JSpac) corresponding to 48 mm (a), 36 mm (b) and 48 mm (c), respectively.

The junction dose distributions were validated experimentally using film dosimetry. For the cases of optimal JSpac shown above, we found a maximum difference between calculated and measured of 3.9% anywhere in the junction region.

### E. Pitch and on‐/off‐axis dependences

**Figure 15:**
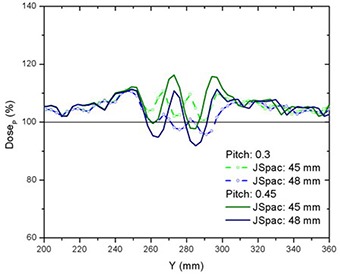
Off‐axis threading effect from junctioning two equal 50 mm width fields for pitches of 0.3 and 0.45, and JSpac of 45 mm and 48 mm (profiles along the central axis of the right PTVJ).

No strong pitch dependence was observed from field matching for the pitches used, with the exception that the off‐axis thread effect was of higher amplitude for larger pitch. The largest thread ripple was observed for a 50 mm field with a pitch of 0.45. Figure 15 shows the off‐axis dose profiles along the central axis of the right PTVJ from matching fields of 50 mm and pitches of 0.3 and 0.45 (JSpac of 45 mm and 48 mm). There are slight differences between on‐ and off‐axis results due to the small variations of the penumbral dose gradients off‐axis (Table 2) and the overlaying thread effect.

## IV. DISCUSSION

In this work we examined field matching with HT. Experiments were based on planning studies after the predicted dose distributions of individual fields were verified by film and ion chamber dosimetry. Agreement between measurement and planned CC penumbral slopes was better than 5%, which is quite reasonable, since this is high dose gradient region, and dosimetric accuracy of Kodak EDR2 films based on the optical density standard deviation ranges from 7% to 15% of their average values.^(23)^

The CC penumbral slope was found to be $\approx D_{P}/\text{field}\;\mathrm{width}$, and gradients were not affected by simultaneously treating one, two or all three PTVs. Thus, penumbral dose gradients at 50% of $D_{P}$ were inversely proportional to the field width and did not depend on the pitch, as shown in Table 2. This could be verified with a simple theoretical calculation of the longitudinal dose profiles along central axis based on a one‐dimensional convolution of an ideal slit‐beam dose profile and an ideal beam intensity distribution.^(24)^

Slightly larger CC penumbral gradients were seen off‐axis than on‐axis. This is related to the beam divergence and it is a result of the same phenomenon that produces thread effect^(21)^ in Helical TomoTherapy. In off‐axis profiles, dose gradients grow faster than on‐axis. For instance, an ideal single rotation on‐axis longitudinal dose profile for a unity pitch is similar to a triangular function.^(21)^ ^,^ ^(24)^ However, off‐axis profiles differ from a triangular shape and the gradients grow faster than linear as previously reported.^(21)^ Similar behavior is expected for smaller pitches as well. The actual field strength delivered by each of the beams varies with the distance to the source. For on‐axis profiles, this distance is kept constant during the whole rotation. For off‐axis profiles, this distance varies, making the field strength weaker when the source is on the far side or making the field width smaller when on the closer side.

**Figure 16:**
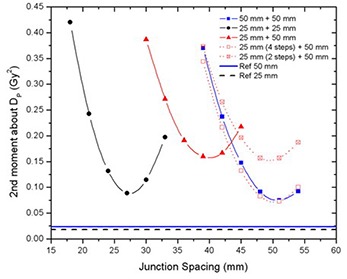
Dose second moments about $D_{P}$ as a function of the JSpac for on‐axis PTVJ from junctioning, equal fields of 50 mm and 25 mm, and different fields of 25 mm and 50 mm.

In the junction region, better homogeneity was obtained for fields of equal width, with larger fields being better than smaller fields, as seen in Fig. 6. Even though junction errors are smaller with larger field widths, there is always an optimal JSpac that allows a satisfactory dose distribution within the junction, even for small fields. Optimal junction spacing was found to be field‐width dependent, being larger for larger fields because of the smaller penumbral dose gradients. The optimal JSpac could be slightly reduced at a cost of increasing the dose to the PTVJs; however, this will increase the minimum dose within the junction. The influence of field width and PTV spacing on the dose in the junction region is summarized in Fig. 16. How widely the dose is spread about the $D_{P}$ is measured by dose second moments.

Junctioning fields of different widths proved to be more problematic. Figures 8, 9 and 14(b) showed that doses spread within a wider range and homogeneous field matching could not be achieved. However, the dose inhomogeneity resulting from junctioning fields of different widths was reduced by breaking PTVs into multiple contiguous PTVs with a modified prescription near the junction to feather the effective junctioning region (see Figs. 12, 13 and 14). Nevertheless, even with the most uniform junction, doses in excess of the prescribed dose were obtained; the average doses to the adjoined PTVs were 4% higher than the average dose obtained using a single continuous field (PTV).

Even though 10 mm fields were not included in this paper, similar reasoning could be applied and an ideal JSpac could be found. Since the penumbral slope would be steeper for 10 mm fields than for 25 or 50 mm fields, a narrower junction region would be obtained.

The objective of this study was to develop methods of producing as homogeneous a dose as possible across a region consisting of multiple contiguous PTVs, although some heterogeneity is normally accepted even in the absence of field junctions. Figures 7 and 10 showed the differential DVHs for on‐axis PTVJs and referential PTVs from matching fields of equal or different widths. The dotted vertical lines enclose the range of dose heterogeneity (−5% and +7% of the prescribed dose) as recommended in the ICRU Report No. 50.^(25)^ The narrowest differential DVH was achieved using equal fields of 50 mm, as shown in Fig. 7(b). Here the dose range extended from −17% to +13% of the prescription, although only 1% of the on‐axis target volume (on‐axis PTVJ) received doses lower than 95% of the $D_{P}$. However, 39% of the PTVJ received more than 107% of the $D_{P}$. This deviation from prescription is larger than the 10% reported by other authors.^(26)^

The pitch had minimal effect on junction dose distributions, with the exception that the off‐axis thread effect was of higher amplitude for larger pitch (Fig. 15). However, this effect can be reduced if specific pitch values are used, as recommended in a recent study.^(21)^ There were slight differences between on‐axis and off‐axis results due to the small variations of the penumbral dose gradients off‐axis (Table 2) and the overlaying thread effect.

The results presented here have limitations, and extrapolation to the clinic must be done with caution. For example, the specific numeric values are valid only in unit density material (water). The principle is valid for all biological materials, but the magnitudes will vary depending on the penumbra, which itself is a function of electron transport in the media and media density. Further, we did not constrain the dose in the SUP‐INF direction as one might do with an organ at risk longitudinally adjacent to the PTV. Thus, in the case of hard constraint optimizations (Kissick et al.^(27)^), the concept presented here remains valid but specific values for optimal JSpac need further investigation.

In spite of its simplicity, this phantom study addresses the principal dosimetric issues which arise when matching two different longitudinally adjacent treatment volumes to form a contiguous treated volume, as might occur when junctioning a recurrence (or new lesion) PTV to a previously treated PTV. Another instance of clinical importance where we see this is when breaking a contiguous PTV into two regions to allow one region to be treated with a small field to gain better PTV/OAR conformance, and the second region to be treated with a larger treatment field to reduce the overall treatment time. A clinical example of where this might be useful is total marrow irradiation. In summary, this study describes the dosimetric challenges that arise when junctioning adjacent PTVs and examines clinically viable solutions.

## V. CONCLUSIONS

We conclude from this study that longitudinal junctioning with HT is possible. Junction errors are smaller with larger field widths. Optimal junction spacing may always be found with acceptable dose distributions. Junctioning using different field widths is more difficult than junctioning fields of the same width. However, breaking PTVs into multiple contiguous PTVs with a modified prescription near the junction will extend the effective junction region, reducing junction dose heterogeneity from matching fields of different widths. Feasibility of longitudinal field junctioning while achieving homogeneous dose distributions with HT extends this technique to clinical situations where dose to contiguous PTVs are planned or delivered independently.
